# Chinese Herbal Medicine Used With or Without Conventional Western Therapy for COVID-19: An Evidence Review of Clinical Studies

**DOI:** 10.3389/fphar.2020.583450

**Published:** 2021-02-26

**Authors:** Shi-Bing Liang, Ying-Ying Zhang, Chen Shen, Chang-Hao Liang, Bao-Yong Lai, Ning Dai, Yu-Qi Li, Zi-Yu Tian, Xiao-Wen Zhang, Yue Jiang, Min Xiong, Ya-Peng Zhang, Ying Zhang, Nicola Robinson, Jian-Ping Liu

**Affiliations:** ^1^ Centre for Evidence-Based Chinese Medicine, Beijing University of Chinese Medicine, Beijing, China; ^2^ The Third Affiliated Hospital, Beijing University of Chinese Medicine, Beijing, China; ^3^ School of Health and Social Care, London South Bank University, London, United Kingdom; ^4^ Institute of Integrated Traditional Chinese Medicine and Western Medicine, Guangzhou Medical University, Guangzhou, China

**Keywords:** traditional Chinese medicine, Chinese herbal medicine, novel coronavirus pneumonia, coronavirus disease 2019, COVID-19, SARS-CoV-2, review, clinical study

## Abstract

**Objective:** To present the evidence of the therapeutic effects and safety of Chinese herbal medicine (CHM) used with or without conventional western therapy for COVID-19.

**Methods:** Clinical studies on the therapeutic effects and safety of CHM for COVID-19 were included. We summarized the general characteristics of included studies, evaluated methodological quality of randomized controlled trials (RCTs) using the Cochrane risk of bias tool, analyzed the use of CHM, used Revman 5.4 software to present the risk ratio (RR) or mean difference (MD) and their 95% confidence interval (CI) to estimate the therapeutic effects and safety of CHM.

**Results:** A total of 58 clinical studies were identified including RCTs (17.24%, 10), non-randomized controlled trials (1.72%, 1), retrospective studies with a control group (18.97%, 11), case-series (20.69%, 12) and case-reports (41.38%, 24). No RCTs of high methodological quality were identified. The most frequently tested oral Chinese patent medicine, Chinese herbal medicine injection or prescribed herbal decoction were: Lianhua Qingwen granule/capsule, Xuebijing injection and Maxing Shigan Tang. In terms of aggravation rate, pooled analyses showed that there were statistical differences between the intervention group and the comparator group (RR 0.42, 95% CI 0.21 to 0.82, six RCTs; RR 0.38, 95% CI 0.23 to 0.64, five retrospective studies with a control group), that is, CHM plus conventional western therapy appeared better than conventional western therapy alone in reducing aggravation rate. In addition, compared with conventional western therapy, CHM plus conventional western therapy had potential advantages in increasing the recovery rate and shortening the duration of fever, cough and fatigue, improving the negative conversion rate of nucleic acid test, and increasing the improvement rate of chest CT manifestations and shortening the time from receiving the treatment to the beginning of chest CT manifestations improvement. For adverse events, pooled data showed that there were no statistical differences between the CHM and the control groups.

**Conclusion:** Current low certainty evidence suggests that there maybe a tendency that CHM plus conventional western therapy is superior to conventional western therapy alone. The use of CHM did not increase the risk of adverse events.

## Introduction

Novel coronavirus pneumonia (NCP), officially named as Coronavirus Disease 2019 (COVID-19) by the World Health Organization (WHO) (World Health Organization, 2020a), is an acute respiratory infectious disease caused by severe acute respiratory syndrome coronavirus 2 (SARS-Cov-2) which has affected the general population. The main symptoms of COVID-19 are fever, cough and fatigue, and may be accompanied by nasal congestion, runny nose, sore throat, diarrhea, or loss of taste and smell anosmia (National Health Commission of the People’s Republic of China, 2020a). In traditional Chinese medicine, COVID-19 is classified within the pestilential (Yibing, 疫病) category. The National Health Commission of the People’s Republic of China has incorporated COVID-19 into the category B infectious diseases as stipulated in the Law of the People’s Republic of China on the Prevention and Control of Infectious Diseases, and carried out prevention and control management following category A infectious diseases. On 11 March 2020, the director-general of World Health Organization (WHO), Dr Tedros Adhanom Ghebreyesus, declared that COVID-19 was now characterized as a pandemic (World Health Organization, 2020b), that is, COVID-19 had spread worldwide, and posed a great challenge and threat to the existing public health resources.

At present, there is no specific and effective therapy for the treatment and prevention of this disease (Chandan et al., 2020; Torequl et al., 2020). Traditional Chinese medicine (TCM) has accumulated thousands of years of experience on the use of Chinese herbal medicine (CHM) to prevent and treat infectious diseases (Jiang 2011). Its success was initially substantiated by modern human clinical research on severe acute respiratory syndrome (SARS) and H1N1 influenza epidemics, suggesting that using historical CHM experience may be a worthwhile approach (Luo et al., 2020). As this current epidemic escalated into a pandemic, the National Health Commission of the People’s Republic of China has released multiple editions of guidelines for the diagnosis and treatment of COVID-19 (hereinafter referred to as GDT of COVID-19). In the third edition (National Health Commission of the People’s Republic of China, 2020b), CHM was recommended for the treatment of COVID-19, and all relevant medical institutions were required to actively encourage of the use of CHM in the treatment of COVID-19. The early application of CHM during the COVID-19 pandemic and appeared to have a potentially beneficial effects. CHM has increasingly shown its potential in the treatment and prevention for infectious diseases, and has received widespread attention.

To further probe the role of CHM used with or without conventional western therapy on the treatment of COVID-19, an evidence-based approach was employed to systematically collate, analyze and evaluate clinical studies on the therapeutic effects and safety of using CHM for COVID-19.

## Materials and Methods

### Inclusion and Exclusion Criteria of Studies

The following criteria were used to identify relevant studies.

Inclusion criteria were as follows: 1) Clinical studies which aimed to evaluate the therapeutic effects and/or safety of CHM used with or without conventional western therapy in patients with COVID-19; 2) There were no limits on the study design and could be randomized controlled trials (RCT), non-randomized controlled trials (non-RCT), cohort studies, case series, case reports or other study designs; 3) Participants were patients diagnosed with COVID-19. Disease severity could be mild, common, severe or critical, as prescribed in the guideline for the diagnosis and treatment of COVID-19 formulated by the National Health Commission of the People’s Republic of China. There was no limitation on participants’ age, gender and their ethnicity, or the setting of the studies; 4) The interventions in the experimental group were CHM and included prescribed herbal decoctions, oral Chinese patent medicines (capsules, tablets or granules) or Chinese herbal medicine injection, or CHM combined with comparators. For controlled clinical studies, comparators could be conventional western therapy or placebo.

Exclusion criteria were: 1) The full text of the studies could not be obtained; 2) Any duplicated articles; 3) Registered clinical studies but had not started or completed; 4) Clinical studies that had been registered and completed but had not published research data, and the data which could not be obtained by contacting the authors; 5) If the registered protocol and the publication(s) were from the same study, the protocol was excluded.

### Retrieval Platforms and Search Strategies of Studies

Studies were retrieved through nine electronic databases including: China National Knowledge Infrastructure (CNKI, as of April 30, 2020), Wanfang Database (from January 1 to April 30, 2020), the China Science Technology Journal Database (VIP, from January 1 to April 30, 2020), SinoMed (from January 1 to April 30, 2020), PubMed (from January 1 to April 30, 2020), Embase (from January 1 to April 30, 2020), BioRxiv (as of April 30, 2020), MedRxiv (as of April 30, 2020), arXiv (as of April 30, 2020) and clinical trial registration platforms (CTRPs) including ClinicalTrials.gov (www.clinicaltrials.gov, as of April 30, 2020) and Chinese Clinical Trial Registry (ChiCTR, www.chictr.org/cn, as of April 30, 2020).

For the databases/CTRPs with COVID-19 thematic platforms, including CNKI and ClinicalTrials.gov, the search was performed directly in the COVID-19 thematic platform. For Wanfang, VIP, SinoMed, PubMed and Embase, search terms were used. The search terms included Xinxing Guanzhuang Bingdu Bing (新型冠状病毒病), Xinguan Feiyan (新冠肺炎), 2019 Guanzhuang Bingdu Bing (2019冠状病毒病), coronavirus disease-19, COVID-19, 2019 novel coronavirus, 2019-nCOV, NCP, Zhongyi (中医), Zhongyao (中药), Caoyao (草药), Tangji (汤剂), Zhongchengyao (中成药), Zhusheji (注射剂), Zhongxiyi Jiehe (中西医结合), Chinese medicine, traditional Chinese medicine, herbal medicine, decoction, patent medicine, injection, integrated Chinese and western medicine. For ChiCTR, title search was carried out using Xinxing Guangzhuang Bingdu (新型冠状病毒) and COVID-19 as search terms. For BioRxiv, MedRxiv and arXiv, title or abstract search was carried out using COVID-19 as search terms. Appendix 1 shows the search strategies for the nine electronic databases and CTRP.

Before submission, we updated the search and included the latest published studies that met the inclusion criteria.

### Study Selection and Data Extraction

Published studies were screened according to the inclusion/exclusion criteria by titles, abstracts and (or) full texts of the published articles. Registered studies were screened according to the inclusion/exclusion criteria by reading the titles and details of registered protocols. SBL, YYZ, CS, CHL, YQL, BYL and ZYT were responsible for the selection of articles.

Excel 2010 was used to provide the data sheets for extraction. Extracted items include first author's name or registered protocol’s ID, study titles, the country in which the study was carried out, study design, characteristics of participants (such as sample size, age, gender, severity of COVID-19, etc.), details of interventions and outcomes, etc. For each included study, two authors independently extracted and checked the data. The inconsistencies were resolved by the two authors through consultation. If any disagreements, a third author (JPL) was consulted. SBL, YYZ, YQL, CS, BYL, ND, YJ, XWZ, CHL, YPZ and MX participated in data extraction in pair.

### Outcomes

Primary outcomes included cure rate, mortality rate and aggravation rate (the change in the disease severity category, or patients were admitted to the ICU, et al.).

Secondary outcomes included the recovery rate or the duration (time to recovery) of main symptoms (including fever, cough and fatigue), negative conversion rate of nucleic acid test for SARS-CoV-2, improvement or recovery of chest CT manifestations, length of hospitalization and adverse events.

For outcomes reported at multiple timepoints, we used the longest reported follow-up timepoint.

### Design of This Review and Data Synthesis

This is an evidence review of clinical studies on the therapeutic effects and safety of CHM used with or without conventional western therapy for COVID-19. Initially, we summarized the general characteristics of the included studies and then the methodological quality of included RCTs was assessed by SBL and YQL using the Cochrane risk of bias tool (Higgins et al., 2011). Subsequently, counts and percentages were applied to analyze the use of CHM. Lastly, we evaluated the therapeutic effects and safety of CHM used with or without conventional western therapy for COVID-19. For studies without control group, such as case series and case reports, we only presented these findings qualitatively as they were not sufficient to probe the therapeutic effects of CHM for COVID-19 due to the absence of control and a high risk of bias in case selection. For studies with control group, we used Cochrane Collaboration Review Manager 5.4 (Revman 5.4) software to conduct meta-analysis of the data. We presented binary data as a risk ratio (RR) with its 95% confidence interval (CI), and continuous data as a mean difference (MD) with its 95% CI. Considering potential sources of clinical heterogeneity, the random-effect model (REM) was used for meta-analysis. We planned to conduct the following subgroup analysis for the primary outcomes if data were available: 1) subgroup analysis based on the severity of COVID-19, to detect whether the therapeutic effects of CHM is related to the severity; 2) subgroup analysis based on the use of CHM with or without conventional western therapy, to detect whether CHM alone or whether CHM plus conventional western therapy is more beneficial for the treatment of COVID-19.

## Results

### Search Results

**FIGURE 1:**
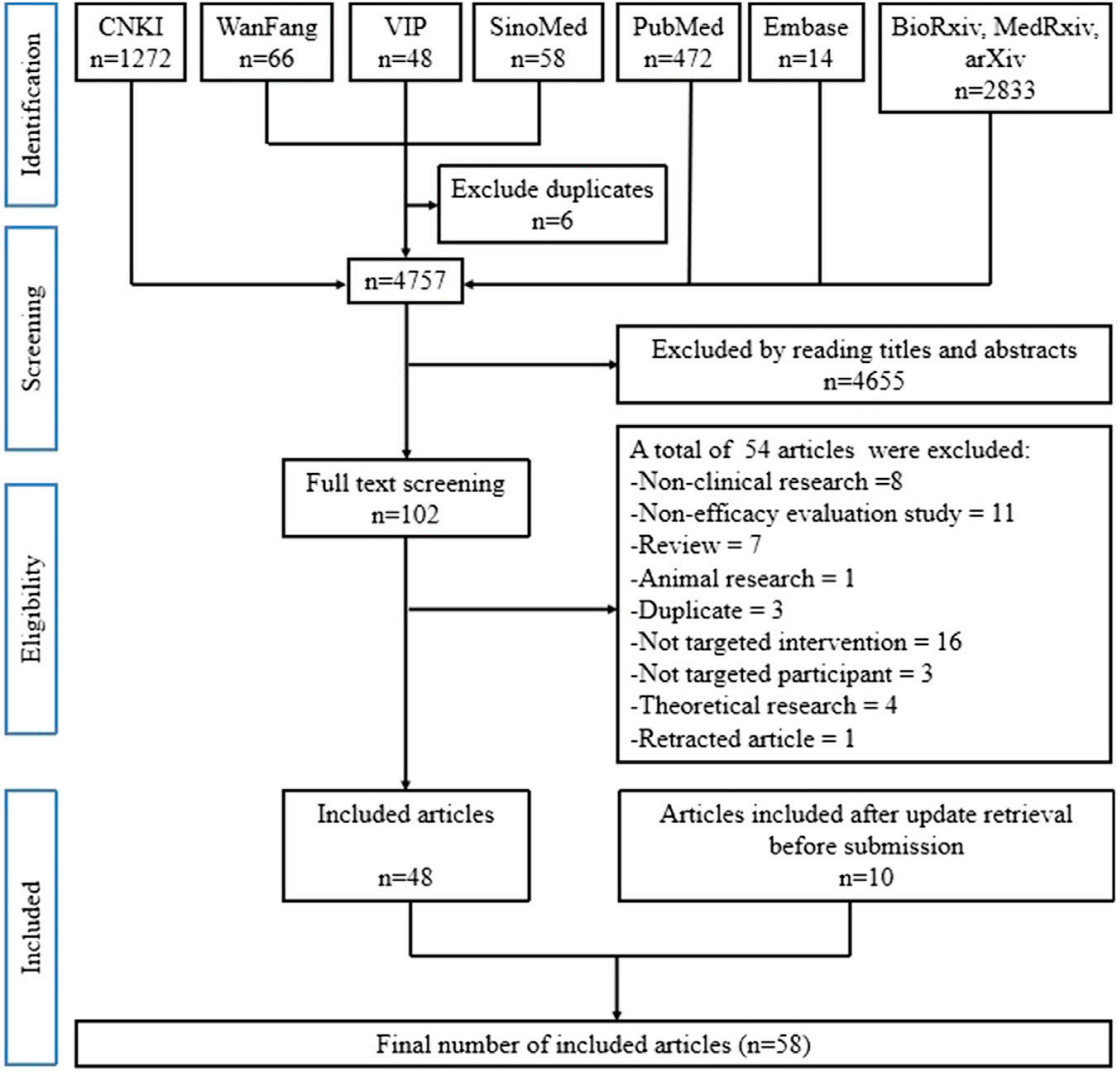
Flow diagram for searching and screening of published articles.

**FIGURE 2:**
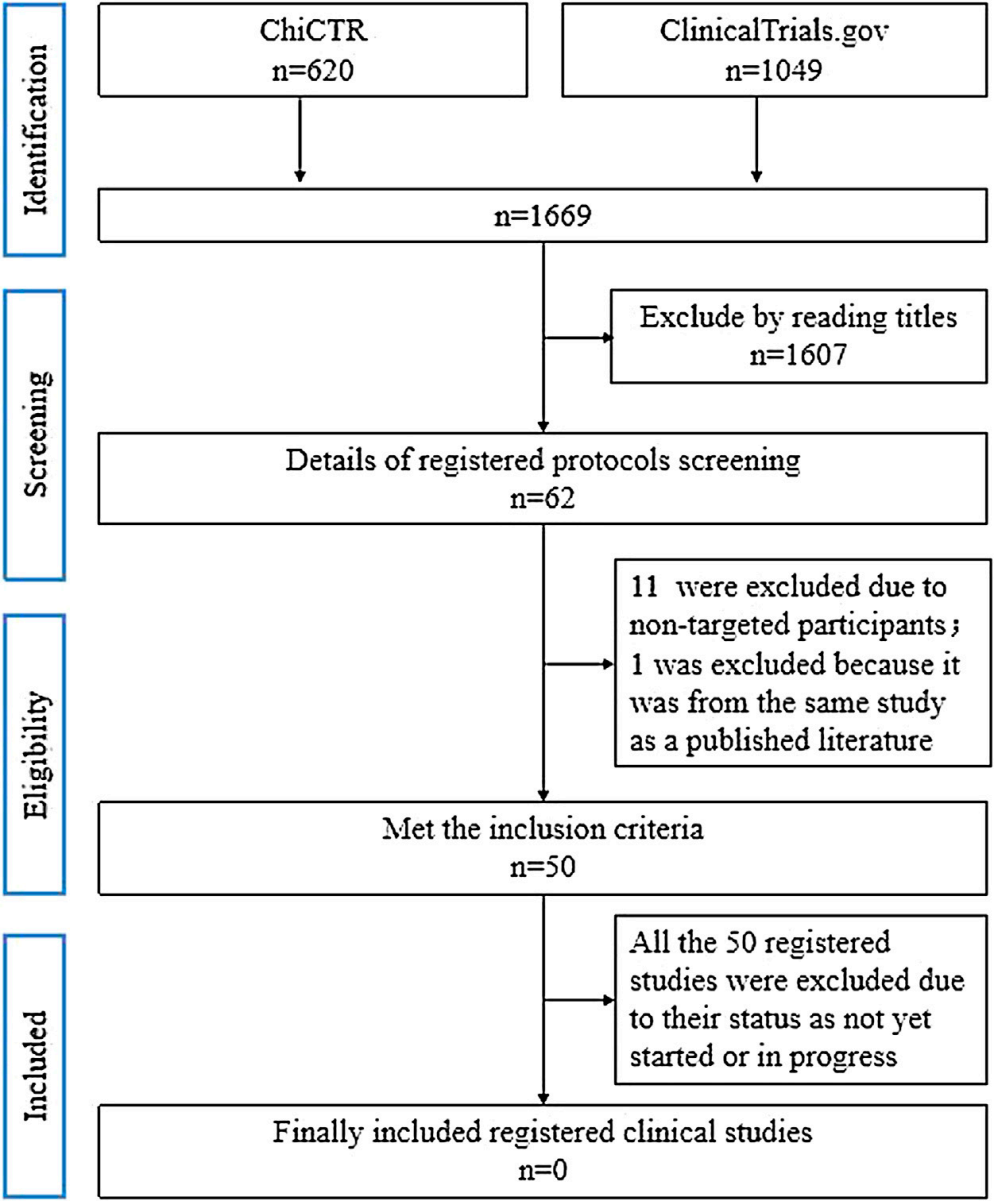
Flow diagram for searching and screening of registered clinical studies.

Figure 1 shows the flow diagram for the searching and screening of published articles. A total of 4763 published articles were retrieved from the above-mentioned nine open electronic databases, of which 102 articles were selected by reading full-texts and 54 were removed for various reasons. Finally, 48 published articles (representing 48 completed studies) met the inclusion criteria. Before submission, we updated the search and included 10 further completed studies that met the inclusion criteria. Figure 2 shows the flow diagram for searching and screening of registered clinical studies. A total of 1669 registered protocols were retrieved from the above-mentioned two CTRPs and 50 registered protocols (50 registered clinical studies) meeting the inclusion criteria. However, all the 50 registered studies were excluded due to their status as ‘not yet started’ or “in progress.”

Therefore, 58 published articles (representing 58 completed studies) were included in our review.

### The Characteristics of Included 58 Clinical Studies

All the 58 clinical studies were conducted in China. Of these, 52 were published in Chinese and six were in English. Among the included studies, 10 (17.24%) were RCTs, one (1.72%) was non-RCT, 11 (18.97%) were retrospective studies with a control group, 12 (20.69%) were case-series, 24 (41.38%) were case-reports.

Of 2773 COVID-19 patients involved in the included studies, 1921 (69.28%) received CHM. The level of severity of COVID-19 involved non-serious (including mild and common) and serious (including severe and critical). Of the included 58 studies, 29 (50.00%) studies included only non-serious patients, 12 (20.69%) studies included only serious patients, 11 (18.97%) included both non-serious and serious patients, and the remaining 6 (10.34%) studies did not report the level of severity of COVID-19.

Of the included 58 studies, 8 (13.79%) involved only the use of CHM, and 51 (87.93%) involved CHM used in combination with conventional western therapy (such as abidor, ganciclovir, lopinavir, oxygen inhalation, nutritional support, etc.). The course of treatment varied from 4 to 15 days.

Table 1 shows the characteristics of the 58 included studies.

**TABLE 1:** The characteristics of included studies of Chinese herbal medicine for COVID-19.

Study ID	Sample size (M/F)	Age (year)	The severity (*) of COVID-19	Type of Chinese herbal medicine	Conventional western therapy (Yes/No)	Course of CHM treatment	Outcomes	Author’s conclusion towards the role of Chinese herbal medicine in the treatment or adjuvant treatment of COVID-19 (positive/negative)
Study type 1: randomized controlled trials (10, 17.24%)								
Yu et al. (2020)	T:82/65, C:89/59	T:48.27±9.56, C:47.25±8.67	Non-serious	Chinese patent medicine	Yes	7 days	②③⑪⑬	Positive
Duan et al. (2020)	T:39/43, C:23/18	T:51.99±13.88, C:50.29±13.17	Non-serious	Chinese patent medicine	Yes	5 days	②④⑤⑥⑬	Positive
Sun et al. (2020)	T:17/15, C:11/14	T:45.4±14.10, C:42.0±11.70	Non-serious	Chinese patent medicine	Yes	14 days	②④⑤⑥⑧⑪	Positive
Fu et al. (2020a)	T:17/15, C:19/14	T:43.26±7.15, C:43.68±6.45	Non-serious	Chinese patent medicine	Yes	10 days	②⑪⑬	Positive
Fu et al. (2020b)	T:19/18, C:19/17	T:45.26±7.25, C:44.68±7.45	Non-serious	Chinese patent medicine	Yes	15 days	①②⑬	Positive
Ding et al. (2020)	T:39/12, C:39/10	T:54.7±21.3, C:50.8±23.5	T: 46 (non-serious) / 5 (serious), C: 11 (non-serious) / 4 (serious)	Prescribed herbal decoction	Yes	10 days	④⑤⑪⑬	Positive
Ye (2020)	T:2/26, C:4/10	T:53.5–69, C:47–67	Serious	Prescribed herbal decoction	Yes	7 days	②③	Positive
Qiu et al. (2020)	T:13/12, C:14/11	T: 53.35±18.35, C:51.32±14.62	Non-serious	Prescribed herbal decoction	Yes	10 days	②⑦⑧⑪	Positive
Zhang et al. (2020a)	T:9/13, C:10/13	T:53.7 ±3.5, C:55.6±4.2	Non-serious	Prescribed herbal decoction	Yes	7 days	⑦⑧⑨⑪⑬	Positive
Wang et al. (2020c)	T:14/10, C:12/11	T:46.8±14.4, C:51.4±17.6	Non-serious	Prescribed herbal decoction	Yes	14 days	②③⑦⑬	Positive
Study type 2: Non-randomized controlled trial (1, 1.72%)								
Xiao et al. (2020)	T:64/36, C:66/34	T:60.90±8.70, C:62.20±7.50	Non-serious	Chinese patent medicine	Yes	14 days	⑦⑧⑨⑪⑬	Positive
Study type 3: Retrospective studies with a control group (11, 18.97%)								
Cheng et al. (2020)	T:26/25, C:27/24	T:55.5±12.3, C:55.8±11.6	Non-serious	Chinese patent medicine	Yes	7 days	②④⑤⑥⑦⑧⑨⑪	Positive
Liu et al. (2020c)	T:21/23, C:16/20	T:50.73, C:51.75	T: 37 (non-serious) / 7 (serious), C: 28 (non-serious) / 8 (serious)	Chinese patent medicine	Yes	7 days	⑪⑬	Positive
Zhang et al. (2020b)	T:10/12, C:12/10	T:25–73, C:19–67	Non-serious	Chinese herbal medicine injection	Yes	7 days	⑩⑪⑬	Positive
Li et al. (2020c)	T:15/15, C:13/17	T:53.600±0.259, C:50.433±0.338	T: 3 (serious)/27(not reported) , C: 2 (serious)/28(not reported)	Prescribed herbal decoction	Yes	Not reported	①②⑦⑧⑪⑬	Positive
Yang et al. (2020c)	T:28/23, C: 24/28	T:61.57±1.84, C:66.35±1.82	Serious	Prescribed herbal decoction + Chinese herbal medicine injection	Yes	Not reported	①③⑪⑫⑬	Positive
Qu et al. (2020a)	T:25/15, C:16/14	T:40.65±8.23, C:39.82±6.40	Non-serious	Chinese patent medicine	Yes	10 days	①⑦⑧⑨⑩⑬	Positive
Xia et al. (2020)	T:17/17, C:6/12	T:54.18±13.08, C:53.67±12.70	T: 27 (non-serious) / 7 (serious) , C: 13 (non-serious) / 4 (serious)	Chinese patent medicine + Chinese herbal medicine injection + prescribed herbal decoction	Yes	5-10 days	①②③⑦⑪⑫⑬	Positive
Yao et al. (2020)	T:16/5, C:12/9	T:57.1±14.0, C:62.4±12.3	Non-serious	Chinese patent medicine	Yes	Not reported	④⑤⑥⑦	Positive
Shi et al. (2020a)	T:26/23, C:10/8	T:47.94±14.46, C:46.72±17.40	T: 41 (non-serious) / 8 (serious) , C: 15 (non-serious) / 3 (serious)	Chinese patent medicine + prescribed herbal decoction	Yes	Not reported	①②⑪⑫	Positive
Yang et al. (2020a)	T:16/10, C:9/14	T:50.35±13.37, C:47.17±16.57	Non-serious	Chinese patent medicine	Yes	7 days	②⑩⑪⑬	Positive
Chen et al. (2020)	T:14/20, C:15/19	T:65.06±10.63, C:64.35±10.34	Non-serious	Chinese patent medicine	Yes	7 days	②④⑤⑥⑦⑧⑨⑪⑫⑬	Positive
Study type 4: Case-series (12, 20.69%)								
Zhang et al. (2020c)	9/15	49.96±12.79 (27-69)	Non-serious	Prescribed herbal decoction	Yes	6-14 days	NA	Positive
Wang et al. (2020d)	52/46	42.70±16.86	87 (non-serious) / 11 (serious)	Prescribed herbal decoction	No	9 days	NA	Positive
Xie et al. (2020a)	8	35–79	Serious	Prescribed herbal decoction	Yes	Not reported	NA	Positive
Li et al. (2020d)	3/3	42–79	Serious	Chinese patent medicine + Chinese herbal medicine injection + prescribed herbal decoction	Yes	Not reported	NA	Positive
Ba et al. (2020)	243/208	43–66	399 (non-serious) / 46 (serious)	Prescribed herbal decoction	Yes	Not reported	NA	Positive
Liu et al. (2020a)	36	NR	Not reported	Prescribed herbal decoction	Yes	14 days	NA	Positive
Huang et al. (2020)	38/33	41.3±16.7	Non-serious	Chinese patent medicine + Chinese herbal medicine injection + prescribed herbal decoction	Yes	Not reported	NA	Positive
Xie et al. (2020b)	27	2–68	Non-serious	Prescribed herbal decoction	Yes	Not reported	NA	Positive
Cheng and Li (2020)	29/25	60.1±16.98 (25–95)	Non-serious	Chinese patent medicine	Yes	8. 0 ± 4. 10 days	NA	Positive
Zhou et al. (2020)	17/23	19–68	Non-serious	Prescribed herbal decoction	Yes	14 days	NA	Positive
Qu et al. (2020b)	23/17	61.2±16.5 (24–79)	Non-serious	Prescribed herbal decoction	Yes	7 days	NA	Positive
Shi et al. (2020c)	15/25	43.9±16.3 (20–94)	32 (non-serious) / 8 (serious)	Prescribed herbal decoction	Yes	Not reported	NA	Positive
Study type 5: Case-reports (24, 41.38%)								
Fu et al. (2020)	1/1	32, 46	Non-serious	Chinese patent medicine	Yes	10/14 days	NA	Positive
Tian et al. (2020)	2/3	24, 28, 36, 40, 49	2 (non-serious) / 3 (serious)	prescribed herbal decoction + Chinese patent medicine	Yes	9 days	NA	Positive
Dong et al. (2020)	1 M	56	Not reported	Prescribed herbal decoction	No	11 day	NA	Positive
Shi et al. (2020b)	2 M	45, 48	Non-serious	Prescribed herbal decoction + Chinese herbal medicine injection	Yes	7/18 days	NA	Positive
Li et al. (2020b)	1/1	35, 36	1 (non-serious) / 1 (serious)	Prescribed herbal decoction	No	4/6 days	NA	Positive
Zhao et al. (2020)	1 F	41	Not reported	Prescribed herbal decoction	Yes	9 days	NA	Positive
He et al. (2020)	2 M	25, 29	Serious	Prescribed herbal decoction	Yes	8/6 days	NA	Positive
Yang and Niu (2020)	1 F	74	Serious	Prescribed herbal decoction	Yes	15 days	NA	Positive
Wang et al. (2020e)	2 M	33, 54	1 (non-serious) / 1 (serious)	Prescribed herbal decoction	Yes	Not reported	NA	Positive
Li et al. (2020e)	1 F	71	Serious	Prescribed herbal decoction	Yes	Not reported	NA	Positive
Feng et al. (2020)	1 F	51	Serious	Prescribed herbal decoction	Yes	15 days	NA	Positive
Xu et al. (2020)	1 M	35	Non-serious	Prescribed herbal decoction	Yes	12 days	NA	Positive
Liu et al. (2020b)	1 F	38	Non-serious	Prescribed herbal decoction	Yes	7 days	NA	Positive
Li et al. (2020f)	2 F	17, 45	Non-serious	Chinese patent medicine + prescribed herbal decoction	No	9 days	NA	Positive
Lin et al. (2020)	1 F	35	Not reported	Prescribed herbal decoction	Yes	12 days	NA	Positive
Hu et al. (2020)	1 F	61	Serious	Chinese patent medicine + prescribed herbal decoction	Yes	11 days	NA	Positive
Wang et al. (2020f)	3/1	19, 32, 63, 63	2 (non-serious) / 2 (serious)	Chinese patent medicine	Yes	Not reported	NA	Positive
Deng et al. (2020)	1 F	39	Serious	Prescribed herbal decoction	Yes	Not reported	NA	Positive
Ni et al. (2020)	1/2	27, 51, 53	Serious	Chinese patent medicine	1 Yes / 2 No	Not reported	NA	Positive
Gao et al. (2020)	1F	42	Non-serious	Chinese patent medicine	No	7 days	NA	Positive
Li et al. (2020a)	1/1	68, 47	Non-serious	Prescribed herbal decoction	No	Not reported	NA	Positive
Lai et al. (2020)	1/2	56, 61, 60	1 (non-serious) / 2 (not reported)	Prescribed herbal decoction	No	6/7 days	NA	Positive
Wang et al. (2020a)	1/1	45, 32	Serious	Chinese herbal medicine injection + prescribed herbal decoction	Yes	12/14days	NA	Positive
Wang et al. (2020b)	1/1	63, 49	Non-serious	Prescribed herbal decoction	Yes	10/14 days	NA	Positive

**Note:** M, male; F, female; T, treatment group involving Chinese herbal medicine; C, controlled group not involving Chinese herbal medicine; Yes, the intervention involved in this study was Chinese herbal medicine combined with conventional western therapy; No, the intervention involved in this trial was Chinese herbal medicines alone, not combined with conventional western therapy; NA, not applicable; Positive, Chinese herbal medicine has benefits on the treatment or adjuvant treatment of COVID-19; negative, Chinese herbal medicine has no benefits on the treatment or adjuvant treatment of COVID-19, or can even make the disease worse.

The severity (*) was classified according to the guidelines for the diagnosis and treatment of COVID-19 released by the National Health Commission of the People’s Republic of China. We divide them into two categories of non-serious (including mild and common) and serious (including severe and critical).

Although the article (Wang et al., 2020c) did not specify the severity of COVID-19, since all participants in this trial were screened from suspected COVID-19 patients, we considered the severity of COVID-19 of these participants as non-serious.

Outcomes: ① cure rate; ② aggravation rate; ③ mortality rate; ④ the recovery rate of fever; ⑤ the recovery rate of cough; ⑥ the recovery rate of fatigue;⑦ the duration of fever; ⑧ the duration of cough; ⑨ the duration of fatigue; ⑩ negative conversion rate of nucleic acid test; ⑪ improvement or recovery of chest CT manifestations; ⑫ Length of hospitalization; ⑬ adverse events.

Although one trial (Yu et al., 2020) reported the outcome of aggravation rate, we did not enrolled the data on this outcome in the statistical analysis due to the inconsistency between the data presented in the table and in the text of the trial’s publication.

### Methodological Quality of RCTs

In terms of the random sequence generation methods of the included 10 RCTs, six RCTs (Fu et al., 2020a; Wang et al., 2020c; Duan et al., 2020; Qiu et al., 2020; Sun et al., 2020; Yu et al., 2020) used random number tables, two trials (Ding et al., 2020; Ye, 2020) used a simple random allocation method and the remaining two RCTs (Zhang et al., 2020a; Fu et al., 2020b) only mentioned random without describing the detailed randomization method. Two RCTs (Wang et al., 2020c; Ye, 2020) performed allocation concealment. Therefore, the risk of selection (allocation) bias was unclear for the majority of the included RCTs due to lack of information on allocation concealment. Due to no trials used blinding to participants and personnel, the performance bias of all the included trials was judged as high-risk. Two RCTs (Wang et al., 2020c; Ye, 2020) performed outcome assessor blinding and the remaining eight RCTs (Fu et al., 2020a; Zhang et al., 2020a; Fu et al., 2020b; Ding et al., 2020; Duan et al., 2020; Qiu et al., 2020; Sun et al., 2020; Yu et al., 2020) did not report relevant information, thus the detection bias for the majority of the included RCTs was judged as unclear-risk. In terms of attrition bias, eight RCTs (Fu et al., 2020a; Zhang et al., 2020a; Wang et al., 2020c; Ding et al., 2020; Duan et al., 2020; Qiu et al., 2020; Sun et al., 2020; Ye, 2020) were assessed as low-risk of bias due to complete outcome data or incomplete outcome data being adequately addressed, two RCTs (Fu et al., 2020b; Yu et al., 2020) were assessed as high-risk due to incomplete outcome data that were not adequately addressed. Two RCTs (Wang et al., 2020c; Ye, 2020) registered the study protocol and reported the registration information. By comparison, we found that there was no selective reporting of outcomes in these two RCTs, so their reporting bias was evaluated as low-risk. Since the protocols or registration information of the other eight included RCTs (Yu et al., 2020; Duan et al., 2020; Sun et al., 2020; Fu et al., 2020a; Fu, et al., 2020b; Ding et al., 2020; Qiu et al., 2020; Zhang et al., 2020a) were not available, the selective reporting of outcomes in these RCTs could not be judged and the reporting bias of these was assessed as unclear-risk. All 10 RCTs reported the comparability of baseline data, so they were assessed as having a low-risk of other bias.

**FIGURE 3:**
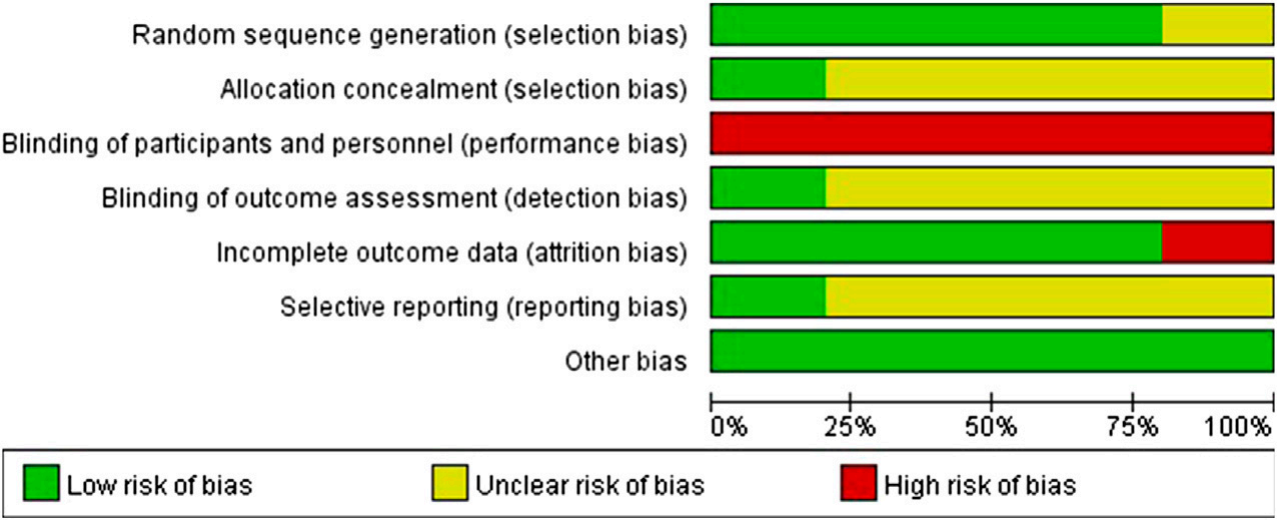
Risk of bias graph of included 10 RCTs.

Figure 3 demonstrates the risk of bias of included 10 RCTs.

### Analysis of the use of CHM

For the type of CHM, 24 (41.38%) studies tested oral Chinese patent medicine, 40 (68.97%) studies tested prescribed herbal decoction, and 7 (12.07%) studies tested Chinese herbal medicine injection.

The top ten CHMs used were Maxing Shigan Tang [麻杏石甘汤, 15.52% (9/58)], Lianhua Qingwen granule/capsule [连花清瘟颗粒/胶囊, 15.52% (9/58)], Xuebijing injection [血必净注射剂, 8.62% (5/58)], Dayuanyin [达原饮, 8.62% (5/58)], Shufeng Jiedu capsule[疏风解毒胶囊, 8.62% (5/58)], Qingfei Paidu Tang [清肺排毒汤, 6.90% (4/58)], Xiaochaihu Tang [小柴胡汤, 6.90% (4/58)], Ganlu Xiaodu Dan [甘露消毒丹, 5.17% (3/58)], Liujunzi Tang [六君子汤, 5.17% (3/58)] and Toujie Quwen granule [透解袪瘟颗粒, 5.17% (3/58)]. Of which, the most frequently used oral Chinese patent medicine, Chinese herbal medicine injection and prescribed herbal decoction were Lianhua Qingwen granule/capsule [连花清瘟颗粒/胶囊], Xuebijing injection [血必净注射剂], and Maxing Shigan Tang [麻杏石甘汤], respectively.

Table 2 lists the CHM used at least twice.

**TABLE 2:** Chinese herbal medicine used twice or more frequently.

The name of Chinese herbal medicine (CHM)	Frequency (N)	Percentage (%)
Type 1 of CHM: Prescribed herbal decoction		
Maxing Shigan Tang [麻杏石甘汤]	9	15.52
Dayuanyin [达原饮]	5	8.62
Qingfei Paidu Tang [清肺排毒汤]	4	6.90
Xiaochaihu Tang [小柴胡汤]	4	6.90
Ganlu Xiaodu Dan [甘露消毒丹]	3	5.17
Liujunzi Tang [六君子汤]	3	5.17
Sanren Tang [三仁汤]	2	3.45
Feiyan No.1 Fang [肺炎1号方]	2	3.45
Xiaoqinglong Tang [小青龙汤]	2	3.45
Wulingsan [五苓散]	2	3.45
Type 2 of CHM: Oral Chinese patent medicine		
Lianhua Qingwen granule/capsule [连花清瘟颗粒/胶囊]	9	15.52
Shufeng Jiedu gapsule[疏风解毒胶囊]	5	8.62
Toujie Quwen granule [透解袪瘟颗粒]	3	5.17
Jinhua Qinggan granule [金花清感颗粒]	2	3.45
Shuanghuanglian oral liquid [双黄连口服液]	2	3.45
Type 3 of CHM: Chinese herbal medicine injection		
Xuebijing injection [血必净注射剂]	5	8.62
Xiyanping injection [喜炎平注射液]	2	3.45
Tanreqing injection [痰热清注射液]	2	3.45
Shenfu injection [参附注射液]	2	3.45
Shengmai injection [生脉注射液]	2	3.45

Note: Frequency refers to the number of included studies using the CHM. Such as, the frequency of Maxing Shigan Tang is 9, which means that nine included studies used Maxing Shigan Tang.

Percentage = (N/58) * 100%

### Therapeutic effects and Safety of CHM in the Treatment or Adjuvant Treatment of COVID-19

#### Analysis for Studies with Control Group

##### Primary Outcomes

###### Cure Rate

Six studies including one RCT (Fu et al., 2020b) and five retrospective studies with a control group (Qu et al., 2020a; Li et al., 2020c; Yang et al., 2020c; Xia et al., 2020; Shi et al., 2020a) reported this outcome. Of which, one study (Shi et al., 2020) was not enrolled into the meta-analysis due to no assessment criteria of cure rate in it's publication. All the other five studies adopted the judgment criteria of the GDT of COVID-19 for cure: 1) the body temperature returned to normal for longer than three days; 2) the respiratory symptoms improved significantly; 3) the pulmonary imaging showed that the inflammation has obviously disappeared; 4) the respiratory pathogenic nucleic acid, (the sampling time interval of two tests was at least 1 day or 24 h), and the results were both negative.

**FIGURE 4:**
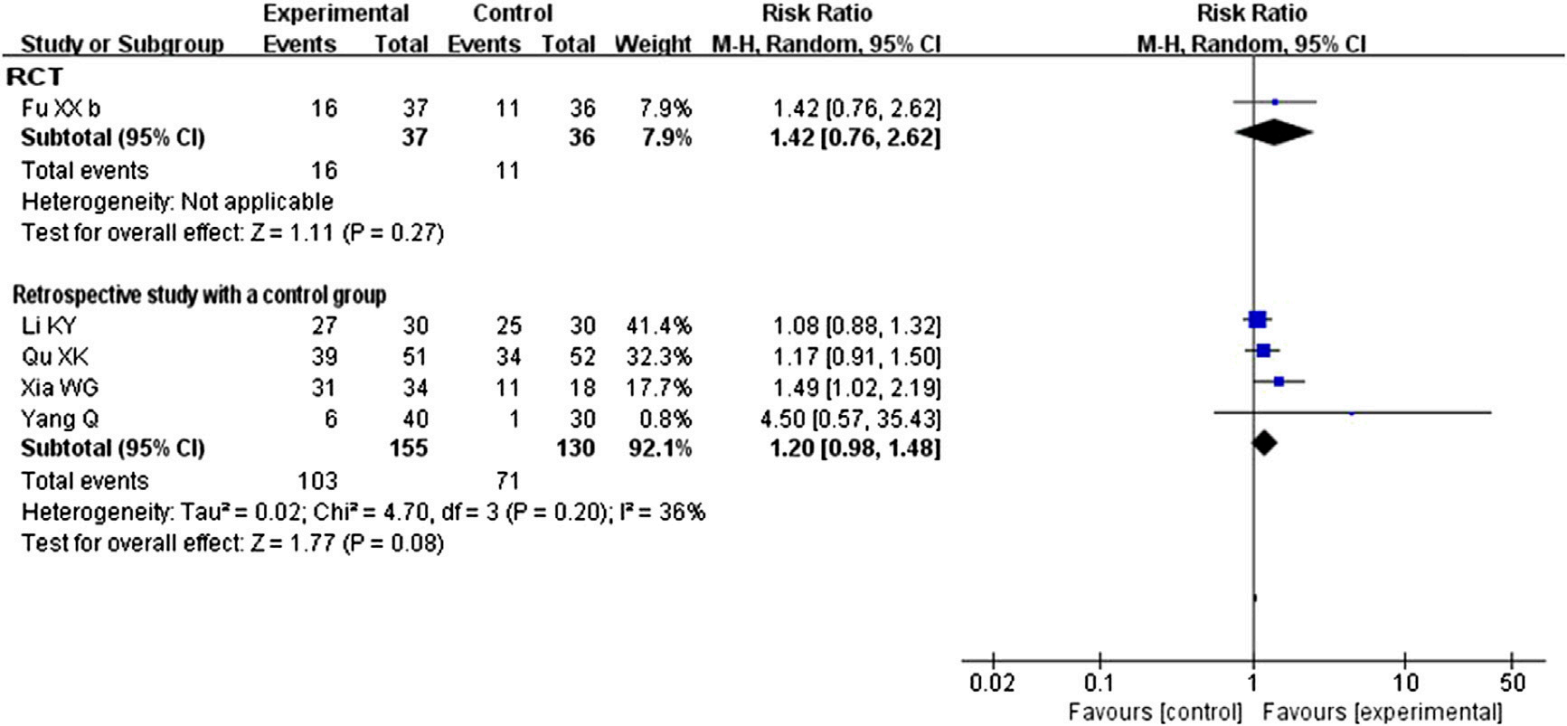
Forest plot of cure rate: CHM plus conventional western therapy vs. conventional western therapy.

All five studies compared CHM plus conventional western therapy with conventional western therapy. After analyzing separately according to the study design, the results (see Figure 4) regardless of RCTs or retrospective studies with a control group showed that there was no statistical difference between the experimental and control groups (RR 1.42, 95% CI 0.76 to 2.62, 1 RCT (Fu et al., 2020b); RR 1.20, 95% CI 0.98 to 1.48, 4 retrospective studies with a control group (Li et al., 2020; Qu et al., 2020a; Xia et al., 2020; Yang et al., 2020c)).

###### Aggravation Rate

**FIGURE 5:**
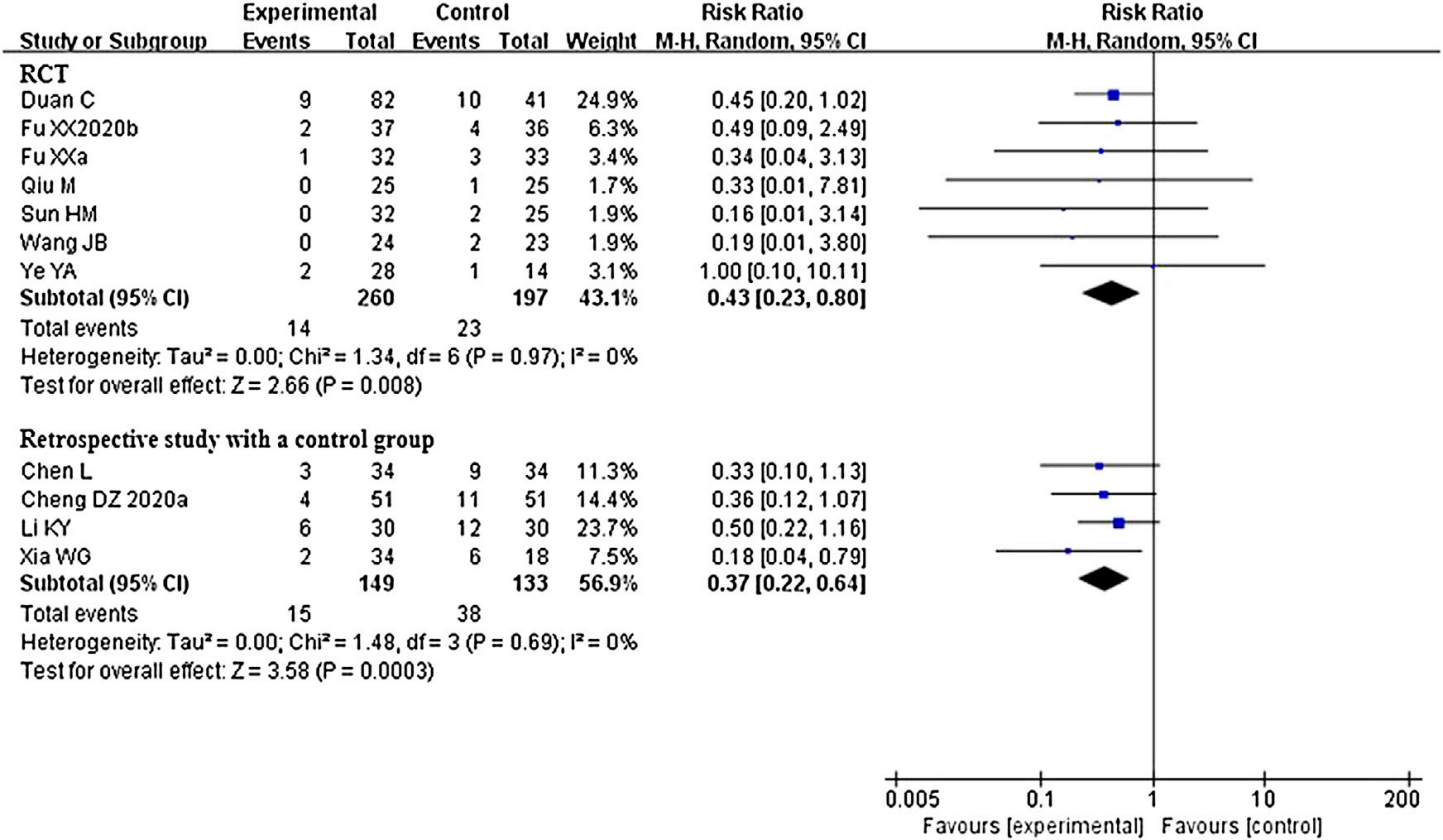
Forest plot of aggravation rate: CHM plus conventional western therapy vs. conventional western therapy.

A total of 14 studies that compared CHM plus conventional western therapy with conventional western therapy reported on this outcome. Of these, two retrospective studies with a control group (Shi et al., 2020a; Yang et al., 2020a) reported that there were no patients who experienced aggravation in either the experimental or control group. Although one study (Yu et al., 2020) reported this outcome in their trial, we did not enrolled the data on this outcome in the statistical analysis due to the inconsistency between the data presented in the table and in the text. After analyzing separately according to the study design of the remaining 11 studies, the results of RCTs or retrospective studies with a control group both showed that CHM plus conventional western therapy was better than conventional western therapy alone in reducing aggravation rate (RR 0.43, 95% CI 0.23 to 0.80, 7 RCTs (Fu et al., 2020a; Wang et al., 2020c; Duan et al., 2020; Qiu et al., 2020; Sun et al., 2020; Ye, 2020; Fu et al., 2020b); RR 0.37, 95% CI 0.22 to 0.64, 4 retrospective studies with a control group (Chen et al., 2020; Cheng et al., 2020; Li et al., 2020c; Xia et al., 2020)). Figure 5 illustrates the details of these results.

###### Mortality Rate

**FIGURE 6:**
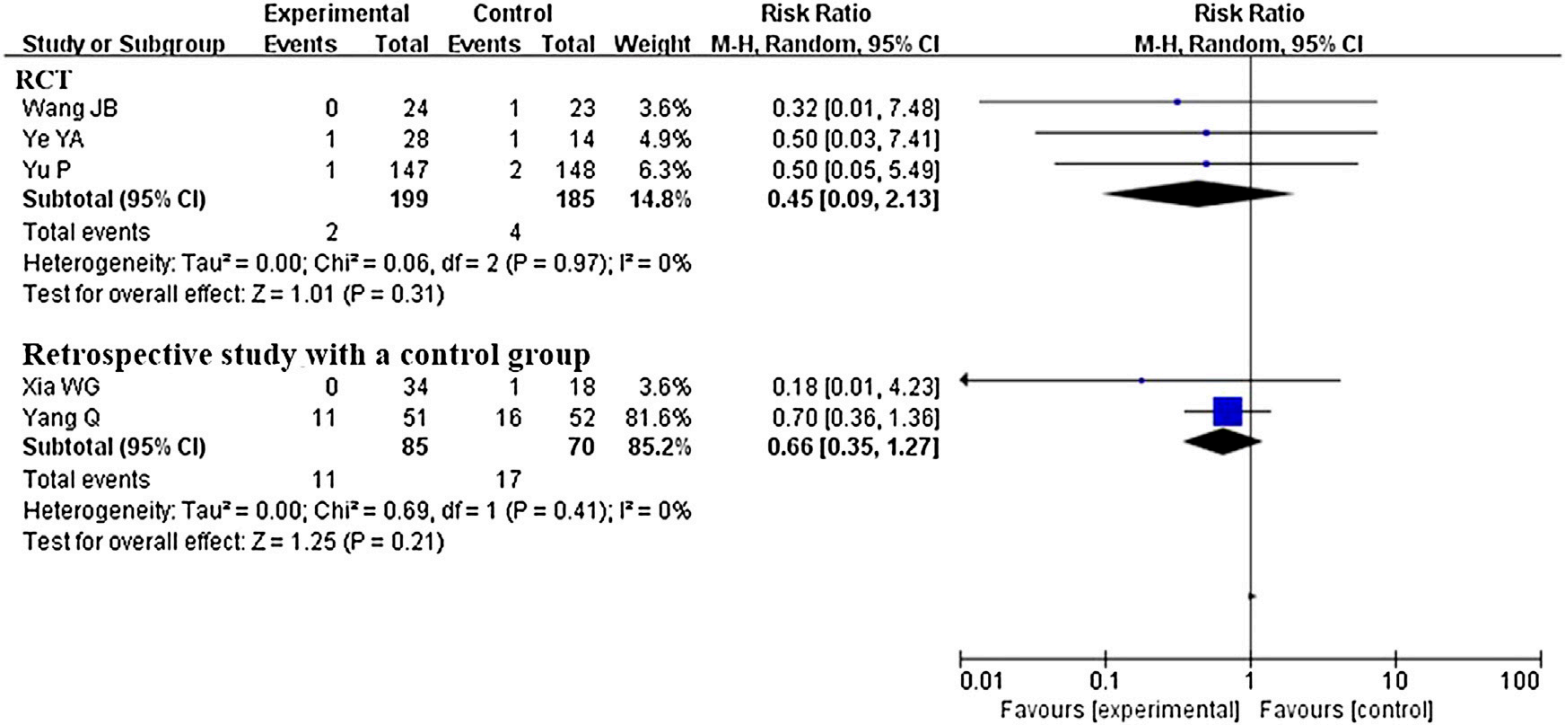
Forest plot of mortality rate: CHM plus conventional western therapy vs. conventional western therapy.

Five studies that compared CHM plus conventional western therapy with conventional western therapy reported this outcome. After analyzing separately according to the study design, the results (see Figure 6) regardless of RCTs or retrospective studies with a control group showed that there was no statistical difference between the experimental and control groups (RR 0.45, 95% CI 0.09 to 2.13, 3 RCTs (Wang et al., 2020c; Ye, 2020; Yu et al., 2020); RR 0.66, 95% CI 0.35 to 1.27, 2 retrospective studies with a control group (Yang et al., 2020c; Xia et al., 2020)).

##### Secondary Outcomes

The results on secondary outcomes are shown in Table 3.

**TABLE 3:** The pooled results of secondary outcomes of CHM used with or without conventional western therapy for COVID-19.

Comparisons and outcomes	Design of the included study	Number of study	Number of participant	The pooled results
Chinese herbal medicine + conventional western therapy vs*.* conventional western therapy				
●The recovery rate of fever	RCT	3	207	RR 1.18, 95% CI 0.91 to 1.54, *I* ^2^ = 64%
	Retrospective study with a control group	3	163	RR 1.34, 95% CI 1.13 to 1.58
●The recovery rate of cough	RCT	3	231	RR 1.36, 95% CI 1.15 to 1.62
	Retrospective study with a control group	3	156	RR 1.82, 95% CI 1.22 to 2.71
●The recovery rate of fatigue	RCT	2	108	RR 1.33, 95% CI 1.03 to 1.71
	Retrospective study with a control group	3	126	RR 1.48, 95% CI 1.14 to 1.93
●The duration of fever	RCT	2	95	MD -2.08 days, 95% CI -2.90 to -1.26, *I* ^2^ = 60%
	Non-RCT	1	200	MD -0.83 days, 95% CI -1.22 to -0.44
	Retrospective study with a control group	6	322	MD -1.54 days, 95% CI -1.82 to -1.26
●The duration of cough	RCT	2	95	MD -2.34 days, 95% CI -3.32 to -1.37, *I* ^2^ = 56%
	Non-RCT	1	200	MD 0.28 days, 95% CI -0.40 to 0.96
	Retrospective study with a control group	4	214	MD -1.68 days, 95% CI -1.92 to -1.43
●The duration of fatigue	RCT	1	45	MD -2.35 days, 95% CI -2.91 to -1.79
	Non-RCT	1	200	MD -0.33 days, 95% CI -0.78 to 0.12
	Retrospective study with a control group	3	136	MD -1.75 days, 95% CI -2.01 to -1.49
●Negative conversion rate of nucleic acid test	Retrospective study with a control group	3	163	RR 1.32, 95% CI 1.05 to 1.66
●The improvement rate of chest CT manifestations	RCT	6	607	RR 1.28, 95% CI 1.10 to 1.49
	Non-RCT	1	200	RR 1.21, 95% CI 1.05 to 1.40
	Retrospective study with a control group	7	484	RR 1.22, 95% CI 1.03 to 1.45, *I* ^2^ = 60%
●The recovery rate of chest CT manifestations	RCT	2	355	RR 1.42, 95% CI 1.00 to 2.02
	Retrospective study with a control group	3	251	RR 1.50, 95% CI 0.97 to 2.31
●The time from receiving treatment to the beginning of chest CT manifestations improvement	Retrospective study with a control group	2	140	MD -2.23 days, 95% CI -2.46 to -2.00
●Length of hospitalization	Retrospective study with a control group	4	290	MD -0.42 days, 95% CI -3.49 to 2.64, *I* ^2^ = 95%
●Adverse events	RCT	3	270	RR 2.06, 95% CI 0.34 to 12.38
	Non-RCT	1	200	RR 1.00, 95% CI 0.21 to 4.84
	Retrospective study with a control group	4	276	RR 0.87, 95% CI 0.26 to 2.93
Chinese herbal medicine vs*.* conventional western therapy		None		

Note: RR, risk ratio; MD, mean difference; CI, confidence interval; RCT, randomized controlled trial; Non-RCT, non-randomized controlled trial.

###### The recovery Rate and the Duration of Main Symptoms (Fever, Cough and Fatigue)

####### a.The recovery rate of main symptoms

A total of six studies including 3 RCTs (Ding et al., 2020; Duan et al., 2020; Sun et al., 2020) and 3 retrospective studies with a control group (Chen et al., 2020; Cheng et al., 2020; Yao et al., 2020) reported the recovery rate of main symptoms. All studies compared CHM plus conventional western therapy with conventional western therapy. Of these, the number of studies that reported the recovery rate of fever, cough and fatigue was six (Chen et al., 2020; Cheng et al., 2020; Ding et al., 2020; Duan et al., 2020; Sun et al., 2020; Yao et al., 2020), six (Chen et al., 2020; Cheng et al., 2020; Ding et al., 2020; Duan et al., 2020; Sun et al., 2020; Yao et al., 2020) and five (Chen et al., 2020; Cheng et al., 2020; Duan et al., 2020; Sun et al., 2020; Yao et al., 2020), respectively.

Regarding studies which explored the recovery rate for fever, after analyzing separately according to the study design, although the pooled data of retrospective studies with a control group showed that CHM plus conventional western therapy was better than conventional western therapy alone (RR 1.34, 95% CI 1.13 to 1.58, 3 retrospective studies with a control group), the pooled result of RCTs showed that there was no statistical difference between the experimental and control groups (RR 1.18, 95% CI 0.91 to 1.54, 3 RCTs, *I* ^2^ = 64%).

Regarding studies which investigated the recovery rate of cough, the results of RCTs or retrospective studies with a control group both showed that CHM in combination with conventional western therapy was superior to conventional western therapy alone (RR 1.36, 95% CI 1.15 to 1.62, 3 RCTs; RR 1.82, 95% CI 1.22 to 2.71, 3 retrospective studies with a control group).

For studies reporting the recovery rate of fatigue following COVID-19, the results regardless of RCTs or retrospective studies with a control group showed that CHM plus conventional western therapy had a higher recovery rate than conventional western therapy alone (RR 1.33, 95% CI 1.03 to 1.71, 2 RCTs; RR 1.48, 95% CI 1.14 to 1.93, 3 retrospective studies with a control group).

####### **b.** The duration (time to recovery) of main symptoms

A total of 11 studies including 4 RCTs (Zhang et al., 2020a; Wang et al., 2020c; Qiu et al., 2020; Sun et al., 2020), 1 non-RCT (Xiao et al., 2020) and 6 retrospective studies with a control group (Qu et al., 2020a; Chen et al., 2020; Cheng et al., 2020; Li et al., 2020c; Xia et al., 2020; Yao et al., 2020) reported the duration of main symptoms and all of them compared CHM plus conventional western therapy with conventional western therapy. Of these, the number of studies that reported the duration of fever, cough and fatigue was ten (Qu et al., 2020a; Zhang et al., 2020a; Chen et al., 2020; Cheng et al., 2020; Li et al., 2020c; Wang et al., 2020c; Qiu et al., 2020; Xia et al., 2020; Xiao et al., 2020; Yao et al., 2020), eight (Qu et al., 2020a; Zhang et al., 2020a; Chen et al., 2020; Cheng et al., 2020; Li et al., 2020c; Qiu et al., 2020; Xiao et al., 2020; Sun et al., 2020) and five (Qu et al., 2020a; Zhang et al., 2020a; Chen et al., 2020; Cheng et al., 2020; Xiao et al., 2020), respectively.

For the duration of fever, one study (Wang et al., 2020c) reported that, the CHM group exhibited a significant improvement in time to fever resolution (*p* = 0.035) compared with the control group. After analyzing separately in light of the other nine studies’ design, the results regardless of RCTs, non-RCT or retrospective studies with a control group showed that CHM plus conventional western therapy was better than conventional western therapy alone in shortening the duration of fever (MD-2.08 days, 95% CI-2.90 to-1.26, 2 RCTs, *I* ^2^ = 60%; MD-0.83 days, 95% CI-1.22 to-0.44, 1 non-RCT; MD-1.54 days, 95% CI-1.82 to-1.26, 6 retrospective studies with a control group).

In shortening the duration of cough, one trial (Sun et al., 2020) reported that CHM group was superior to conventional western therapy alone in shortening the duration of cough (P < 0.5). After analyzing separately based on the studys' design, the results regardless of RCTs or retrospective studies with a control group showed that CHM plus conventional western therapy was superior to conventional western therapy alone (MD-2.34 days, 95% CI-3.32 to-1.37, 2 RCTs, *I* ^2^ = 56%; MD-1.68 days, 95% CI-1.92 to-1.43, 4 retrospective studies with a control group). However, the results from one non-RCT showed that there was no statistical difference between the experimental and control groups (MD 0.28 days, 95% CI -0.40 to 0.96, 1 non-RCT).

Regarding those studies reporting the duration of fatigue as secondary outcome, both RCTs and retrospective studies with a control group showed better effects for the CHM plus conventional western therapy when compared with conventional western therapy alone (MD -2.35 days, 95% CI-2.91 to-1.79, 1 RCT; MD-1.75 days, 95% CI-2.01 to-1.49, 3 retrospective studies with a control group). However, the result from one non-RCT showed that there was no statistical difference between the two groups (MD-0.33 days, 95% CI-0.78 to 0.12, 1 non-RCT).

###### Negative Conversion Rate of Nucleic Acid Test for SARS-Cov-19

A total of three retrospective studies with a control group (Qu et al., 2020a; Yang et al., 2020a; Zhang et al., 2020b) reported this outcome and all compared CHM plus conventional western therapy with conventional western therapy. Pooled data from 3 studies showed that CHM in combination with conventional western therapy was superior to conventional western therapy alone (RR 1.32, 95% CI 1.05 to 1.66) in improving the negative conversion rate of nucleic acid test for SARS-Cov-19.

###### Improvement or Recovery of Chest CT Manifestations

A total of 16 studies (Yu et al., 2020; Sun et al., 2020; Fu et al., 2020a; Ding et al., 2020; Xiao et al., 2020; Cheng et al., 2020; Liu et al., 2020c; Zhang et al., 2020b; Li et al., 2020c; Yang et al., 2020c; Xia, et al., 2020; Shi et al., 2020a; Yang et al., 2020a; Qiu et al., 2020; Zhang et al., 2020a; Chen et al., 2020) reported this outcome and all compared CHM plus conventional western therapy with conventional western therapy.

Of these, 14 studies (Fu et al., 2020a; Shi et al., 2020a; Yang et al., 2020a; Zhang et al., 2020a; Zhang et al., 2020b; Chen et al., 2020; Cheng et al., 2020; Yang et al., 2020c; Ding et al., 2020; Qiu et al., 2020; Sun et al., 2020; Xia et al., 2020; Xiao et al., 2020; Yu et al., 2020) the improvement rate of chest CT manifestations (improvement rate = the number of patients with improvement of chest CT manifestations/the total number of patients in experimental or control group × 100%). After analyzing separately according to the study design, the results regardless of RCTs, non-RCT or retrospective studies with a control group showed that CHM plus conventional western therapy was better than conventional western therapy alone in increasing the improvement rate of chest CT manifestations (RR1.28, 95% CI 1.10 to 1.49, 6 RCTs; RR 1.21, 95% CI 1.05 to 1.40, 1 non-RCT; RR 1.22, 95% CI 1.03 to 1.45, 7 retrospective studies with a control group). Five studies (Fu et al., 2020a; Yu et al., 2020; Chen et al., 2020; Yang et al., 2020c, Liu et al., 2020c) reported the recovery rate of chest CT manifestations (recovery rate = the number of patients with recovery of chest CT manifestations / the total number of patients in experimental or control group × 100%). After analyzing separately according to the studys' design, the results demonstrated that there was no statistical difference between the two groups in increasing the recovery rate of chest CT manifestations (RR 1.42, 95% CI 1.00 to 2.02, 2 RCTs; RR 1.50, 95% CI 0.97 to 2.31, 3 retrospective studies with a control group).

The other two retrospective studies with a control group (Li et al., 2020c; Liu et al., 2020c) reported the time from receiving the treatment to the beginning of chest CT manifestations improvement and the pooled analysis from the two studies showed that CHM plus conventional western therapy was superior to conventional western therapy alone in shortening the time (MD-2.23 days, 95% CI-2.46 to -2.00, two retrospective studies with a control group).

###### Length of Hospitalization

A total of four retrospective studies with a control group (Shi et al., 2020a; Yang et al., 2020c; Qiu et al., 2020; Xia et al., 2020) reported length of time in hospital as an outcome. All four studies compared CHM plus conventional western therapy with conventional western therapy. The pooled analysis from the four studies showed that there was no statistical difference between the experimental and control groups (MD -0.42 days, 95% CI -3.49 to 2.64, *I* ^2^ = 95%) in shortening the length of hospitalization.

###### Adverse Events

A total of 16 studies reported this outcome and all compared CHM plus conventional western therapy with conventional western therapy. Of these, eight studies (Fu et al., 2020a; Yang et al., 2020a; Zhang et al., 2020a; Fu et al., 2020b; Chen et al., 2020; Liu et al., 2020c; Xia et al., 2020; Yu et al., 2020) reported that no adverse events occurred in either the experimental or control group. Pooled data from the other eight studies (Qu et al., 2020a; Li et al., 2020c; Wang et al., 2020c; Yang et al., 2020c; Zhang et al., 2020c; Ding et al., 2020; Duan et al., 2020; Xiao et al., 2020) showed that there was no statistical difference between the experimental and control groups (RR 2.06, 95% CI 0.34 to 12.38, three RCTs (Duan et al., 2020; Ding et al., 2020; Wang, et al., 2020c); RR 1.00, 95% CI 0.21 to 4.84, one non-RCT (Xiao et al., 2020); RR 0.87, 95% CI 0.26 to 2.93, four retrospective studies with a control group (Zhang et al., 2020a; Li et al., 2020c; Yang, et al., 2020c; Qu et al., 2020a)). The adverse events reported in these eight studies were mild abdominal pain, diarrhea, nausea, vomiting and drug allergy, et al.

##### Subgroup Analysis

As all controlled studies compared CHM plus conventional western therapy with conventional western therapy, we failed to perform the subgroup analysis based on the use of CHM with or without conventional western therapy. Therefore, we only conducted the subgroup analysis based on the level of severity of COVID-19 (non-serious, serious or a mix of non-serious and serious) for primary outcomes.

With regard to cure rate, although a pooled data of five studies that reported this outcome showed that CHM plus conventional western therapy was superior to conventional western therapy in improving it (RR 1.21, 95% CI 1.01 to 1.45), the results (see Supplement-Figure 1) of the subgroup analysis based on the level of severity of COVID-19 showed that there was no statistical difference between the experimental and control groups (RR 1.69, 95% CI 0.72 to 3.92, two studies (Qu et al., 2020a; Fu et al., 2020b) involving 143 non-serious patients; RR 1.17, 95% CI 0.91 to 1.50, one study (Yang et al., 2020c) involving 103 serious patients; RR 1.23, 95% CI 0.87 to 1.72, two studies (Li et al., 2020c; Xia et al., 2020) involving 112 patients, a mix of non-serious and serious, *I* ^2^ = 62%).

Regarding aggravation rate, a total of 11 studies (Fu et al., 2020b; Duan et al., 2020; Sun et al., 2020; Fu et al., 2020a; Ye, 2020; Cheng et al., 2020; Li, et al., 2020c; Xia et al., 2020; Qiu et al., 2020; Chen et al., 2020; Wang et al., 2020c) that reported this outcome were used to conduct meta-analysis, and the results (see Supplement Figure-2) from the 11 studies showed that CHM plus conventional western therapy was better than conventional western therapy alone in reducing aggravation rate (RR 0.40, 95% CI 0.26 to 0.59). Of which, seven studies (Duan et al., 2020; Sun et al., 2020; Fu et al., 2020a; Cheng et al., 2020, Qiu et al.,2020 ; Chen et al., 2020; Wang et al., 2020c) included only patients with non-serious COVID-19, and pooled data from the seven studies showed that CHM plus conventional western therapy was better than conventional western therapy (RR 0.37, 95% CI 0.22 to 0.63, seven studies). One study (Ye, 2020) included only patients with serious COVID-19, the results showed that there was no statistical difference between the experimental and control groups (RR 1.00, 95% CI 0.10 to 10.11, one study). The remaining three studies (Fu et al., 2020b; Li et al., 2020c; Xia et al., 2020) included both non-serious patients and serious patients with COVID-19, and the results from the three studies showed a lower aggravation rate in the experimental group compared with the control group (RR 0.40, 95% CI 0.21 to 0.79, three studies).

For mortality rate, a total of five studies (Wang et al., 2020c; Yang et al., 2020c; Xia et al., 2020; Ye, 2020; Yu et al., 2020) were included, and pooled data from five studies showed that there was no statistical difference between the experimental and control groups (RR 0.62, 95% CI 0.34 to 1.14) in reducing mortality rate. The results (see Supplement-Figure 3) of the subgroup analysis based on the level of severity of COVID-19 showed that there was also no statistical difference between the two groups (RR 0.43, 95% CI 0.06 to 2.86, two study (Wang et al., 2020c; Yu et al., 2020) involving 342 non-serious patients; RR 0.69, 95% CI 0.36 to 1.31, two studies (Yang et al., 2020c; Ye, 2020) involving 145 serious patients; RR 0.18, 95% CI 0.01 to 4.23, one study (Xia et al., 2020) involving 52 patients, a mix of non-serious and serious).

#### Analysis of Case Series and Case Reports

A total of 12 case series and 24 case reports were included in our review. Of which, one case series and 7 case reports involving 111 patients only used CHM, and 11 case series and 19 case reports involving 828 patients used CHM plus conventional western therapy. The authors of the 36 articles concluded that CHM with or without conventional western therapy was beneficial for the treatment of COVID-19.

With regard to 111 patients who received CHM treatment for a period of time from 4 to 11 days, one case series and one case report involving 100 patients reported that 42 patients were cured (42/100), 7 case reports involving 13 patients reported that 13 patients were negative for nucleic acid test (13/13), one case series and 6 case reports involving 54 patients reported that 30 patients with the recovery of fever (30/54), one case series and one case report involving 71 patients reported that 17 patients with the recovery of cough (17/71), one case series involving 75 patients reported that 20 patients with the recovery of fatigue (20/75), one case series and 5 case reports involving 96 patients reported that 87 patients (87/96) showed improvement or recovery of chest CT manifestations.

For 828 patients who received CHM plus conventional western therapy for a period of time from 6 to 15 days, 4 case series and 6 case reports involving 641 patients reported that 561 patients were cured (561/641), 6 case series and 16 case reports involving 182 patients reported that 179 patients were negative for nucleic acid test (179/182), 5 case series and 13 case reports involving 271 patients reported that 258 patients with the recovery of fever (258/271), 5 case series and 3 case reports involving 437 patients reported that 284 patients with the recovery of cough (284/437), 5 case series and 2 case reports involving 327 patients reported that 212 patients with the recovery of fatigue (212/327), and 3 case series and 11 case reports involving 525 patients reported that 483 patients (483/525) showed improvement or recovery of chest CT manifestations. In addition, there were 3 case series which reported adverse events. Of these, 2 case series reported that no adverse events occurred, and the remaining reported that seven patients with the treatment of CHM plus conventional western therapy experienced adverse events including vomiting (4), dizziness 2) and rash (1).

## Discussion

Although RCT is the gold standard to evaluate the therapeutic effects of interventions, it cannot answer all the important questions about a given intervention (Black, 1996). Considering the characteristics of sudden acute infectious diseases and the practical problems of ethics and informed consent, the implementation of RCT faces more challenges under conventional medical conditions (Yang et al., 2020b). Many questions in medical research are investigated in observational studies having a role in research into the benefits and harms of medical interventions (Black, 1996; Glasziou et al., 2004), having an important reference for the preliminary evaluation of the therapeutic effects of CHM and clinical decision-making. In this case, other types of studies (e.g., non-RCT, retrospective studies, case-series) were included in our review.

### Summary of the Main Findings

A total of 58 clinical studies whose purpose were to evaluate the therapeutic effects of CHM used with or without conventional western therapy for COVID-19 were included. The included studies involved RCTs, non-RCT, retrospective studies with a control group, case-series and case-reports. In total the studies involved 2773 COVID-19 patients, 1921 (69.28%) of them received CHM. The severity of COVID-19 varied from non-serious (mild and common) and serious (severe and critical). Most of the studies used a combination of CHM and conventional western therapy. Analysis of the frequency of different CHM indicated that the most frequently used oral Chinese patent medicine, Chinese herbal medicine injection and prescribed herbal decoction were Lianhua Qingwen granule/capsule, Xuebijing injection, and Maxing Shigan Tang, respectively.

This review suggested that CHM in combination with conventional western therapy appeared better than conventional western therapy alone in reducing aggravation rate, increasing the recovery rate or shortening the duration of main symptoms (fever, cough and fatigue), improving the negative conversion rate of nucleic acid test, increasing the improvement rate of chest CT manifestations and shortening the time from receiving the treatment to the beginning of chest CT manifestations improvement. For the primary outcomes, subgroup analyses were conducted based on the level of severity of COVID-19 and suggested that CHM in combination with conventional western therapy had more significant effect than conventional western therapy in reducing aggravation rate for non-serious patients.

In terms of reducing mortality rate and shortening the length of hospitalization, there was no statistical difference between the CHM combined conventional western therapy group and the conventional western therapy group. Although some studies have reported adverse events (e.g., mild abdominal pain, diarrhea, nausea and vomiting) in the CHM plus conventional western therapy group, but there was also no statistical difference between the experimental and control groups. This suggests that the use of CHM did not increase the risk of adverse events.

Although in this review there were no pooled results for CHM used alone from controlled studies for COVID-19, one case-series and seven case-reports that were included reported that CHM alone may play a positive therapeutic role in the treatment of COVID-19.

### Strengths and Limitations

This review systematically collected the evidence from clinical studies whose purpose was to evaluate the therapeutic effects and safety of CHM with or without conventional western therapy for COVID-19. Relevant clinical studies were analyzed from the aspects of general characteristics, quality assessment, analysis of the use of CHM, therapeutic effects and safety of CHM for COVID-19 patients, providing important evidence for future related research.

However, this review did not summarize the specific administration methods of CHM in all the included studies, especially considering the complexity of prescribed herbal decoction use, which may require further specific research in the future. Therefore, this review cannot be directly used to guide clinical practice. In addition, all included studies were conducted in China, whether this evidence is equally applicable to other countries outside China needs further international study.

### Implications for Further Research

The benefits for the use of CHM for COVID-19 needs to be verified by more rigorous designed and implemented clinical trials, especially randomized controlled trials. The following points should be noted when conducting relevant RCTs: 1) Clear reporting of random allocation and random concealment; 2) Application of blinding to participants, personnel (doctors), outcome evaluators and statistical analysts; 3) Design and register the study protocol; 4) Definition of important outcomes, such as time to cure, aggravation and mortality; 5) Selection of CHM: considering the difficulty in the use of herbal decoction (e.g., dosage of herbal medicien, especially about its use outside China), we suggest that trials of oral Chinese patent medicine or Chinese herbal medicine injection should be given priority to verify the therapeutic effects and safety of these two, so as to find safe, effective and convenient medications to cure more COVID-19 patients as soon as possible. Unfortunately, in our this research, we did not to perform subgroup analysis on oral Chinese patent medicine, Chinese herbal medicine injection and prescribed Chinese herbal medicine decoction.

## Conclusion

Current low certainty evidence suggests that there maybe a tendency that CHM plus conventional western therapy is superior to conventional western therapy alone. The use of CHM did not increase the risk of adverse events.

## Appendix 1Search strategies for the nine electronic databases and clinical trial registration platforms (CTRP).

**Table:**

Databases/CTRP	Search strategy	Time limit
CNKI	Since CNKI has set up a thematic platform for COVID - 19, the "treatment" section of the platform was selected for manual retrieval	As of April 30, 2020
VIP	#1: M = Xinxing Guangzhuang Bingdu Bing(新型冠状病毒病) OR Xinguan Feiyan (新冠肺炎) OR 2019 Guanzhuang Bingdu (2019冠状病毒病) OR COVID-19 OR 2019-nCOV OR NCP	From January 1 to April 30, 2020
	#2: M = Zhongyi (中医) OR Zhongyao (中药) OR Caoyao (草药) OR Tangji (汤剂) OR Zhongchengyao (中成药) OR Zhusheji (注射剂) OR Zhongxiyi Jiehe (中西医结合)	
	#3: #1 AND #2	
Wanfang	#1: Major Topic: "Xinxing Guangzhuang Bingdu Bing (新型冠状病毒病)" + "Xinguan Feiyan (新冠肺炎)" + " 2019 Guanzhuang Bingdu Bing (2019冠状病毒病)" + "COVID-19" + "2019-nCOV" + "NCP"	From January 1 to April 30, 2020
	#2: Major Topic: "Zhongyi (中医)" + "Zhongyao (中药)" + "Caoyao (草药)" + "Tangji (汤剂)" + "Zhongchengyao (中成药)" + "Zhusheji (注射剂)" + "Zhongxiyi Jiehe (中西医结合)"	
	#3: #1 AND #2	
SinoMed	#1: ("Xinxing Guangzhuang Bingdu Bing (新型冠状病毒病)"[标题:智能] OR "Xinguan Feiyan (新冠肺炎)"[标题:智能] OR "2019 Guanzhuang Bingdu Bing (2019冠状病毒病)"[标题:智能] OR "COVID-19"[标题:智能] OR "2019-nCOV"[标题:智能] OR "NCP"[标题:智能])	From January 1 to April 30, 2020
	#2: ("Zhongyi (中医)"[标题:智能] OR "Zhongyao (中药)"[标题:智能] OR "Caoyao (草药)"[标题:智能] OR "Tangji (汤剂)"[标题:智能] OR "Zhongchengyao (中成药)"[标题:智能] OR "Zhusheji (注射剂)"[标题:智能] OR "Zhongxiyi Jiehe (中西医结合)"[标题:智能])	
	#3: #1 AND #2	
PubMed	(((Corona virus disease-19 OR COVID-19 OR 2019 novel coronavirus OR 2019-nCOV OR NCP[MeSH Major Topic]) AND (Chinese medicine OR traditional Chinese medicine OR herbal medicine OR decoction OR patent medicine OR injection OR integrated Chinese and western medicine[MeSH Major Topic]) AND ("2020/01/01"[Date-Publication] : "2020/04/30"[Date-Publication])	From January 1 to April 30, 2020
Emabse	#1: ab,ti: corona virus disease-19 OR COVID-19 OR 2019 novel coronavirus OR 2019-nCOV OR NCP	From January 1 to April 30, 2020
	#2: ab,ti: Chinese medicine OR traditional Chinese medicine OR herbal medicine OR decoction OR patent medicine OR injection OR integrated Chinese and western medicine	
	#3: #1 AND #2	
ChiCTR	Title search was carried out using Xinxing Guangzhuang Bingdu (新型冠状病毒) and COVID-19 as search terms	As of April 30, 2020
ClinicalTrials.gov	Searched in covid-19 special registration section	As of April 30, 2020
BioRxiv, MedRxiv, arxiv	Title or abstract search was carried out using COVID-19 as search terms	As of April 30, 2020
