# Supplementary material for: Chinese Herbal Medicine Used With or Without Conventional Western Therapy for COVID-19: An Evidence Review of Clinical Studies
Source: Front Pharmacol. 2021 Feb 26;11:583450. doi: 10.3389/fphar.2020.583450 (PMC7953048; doi:10.3389/fphar.2020.583450)
Supplement: Supplementary file 1 [file datasheet1.pdf]

Supplement

Study or Subgroup	Experimental Events	Experimental Total	Control Events	Control Total	Weight	Risk Ratio M-H, Random, 95% CI
**1.2.1 Mild or common type (Non-serious)**						
Fu XXb	16	37	11	36	7.9%	1.42 [0.76, 2.62]
Qu XK	6	40	1	30	0.8%	4.50 [0.57, 35.43]
**Subtotal (95% CI)**		**77**		**66**	**8.7%**	**1.69 [0.72, 3.92]**
Total events	22		12			
Heterogeneity: Tau² = 0.12; Chi² = 1.20, df = 1 (P = 0.27); I² = 17%						
Test for overall effect: Z = 1.21 (P = 0.23)						
**1.2.2 Severe or acute severe type (Serious)**						
Yang Q	39	51	34	52	32.3%	1.17 [0.91, 1.50]
**Subtotal (95% CI)**		**51**		**52**	**32.3%**	**1.17 [0.91, 1.50]**
Total events	39		34			
Heterogeneity: Not applicable						
Test for overall effect: Z = 1.23 (P = 0.22)						
**1.2.3 Mix type (Mix of non-serious and serious)**						
Li KY	27	30	25	30	41.4%	1.08 [0.88, 1.32]
Xia WG	31	34	11	18	17.7%	1.49 [1.02, 2.19]
**Subtotal (95% CI)**		**64**		**48**	**59.1%**	**1.23 [0.87, 1.72]**
Total events	58		36			
Heterogeneity: Tau² = 0.04; Chi² = 2.61, df = 1 (P = 0.11); I² = 62%						
Test for overall effect: Z = 1.17 (P = 0.24)						
**Total (95% CI)**		**192**		**166**	**100.0%**	**1.21 [1.01, 1.45]**
Total events	119		82			
Heterogeneity: Tau² = 0.01; Chi² = 5.29, df = 4 (P = 0.26); I² = 24%						
Test for overall effect: Z = 2.07 (P = 0.04)						
Test for subgroup differences: Chi² = 0.67, df = 2 (P = 0.72), I² = 0%						

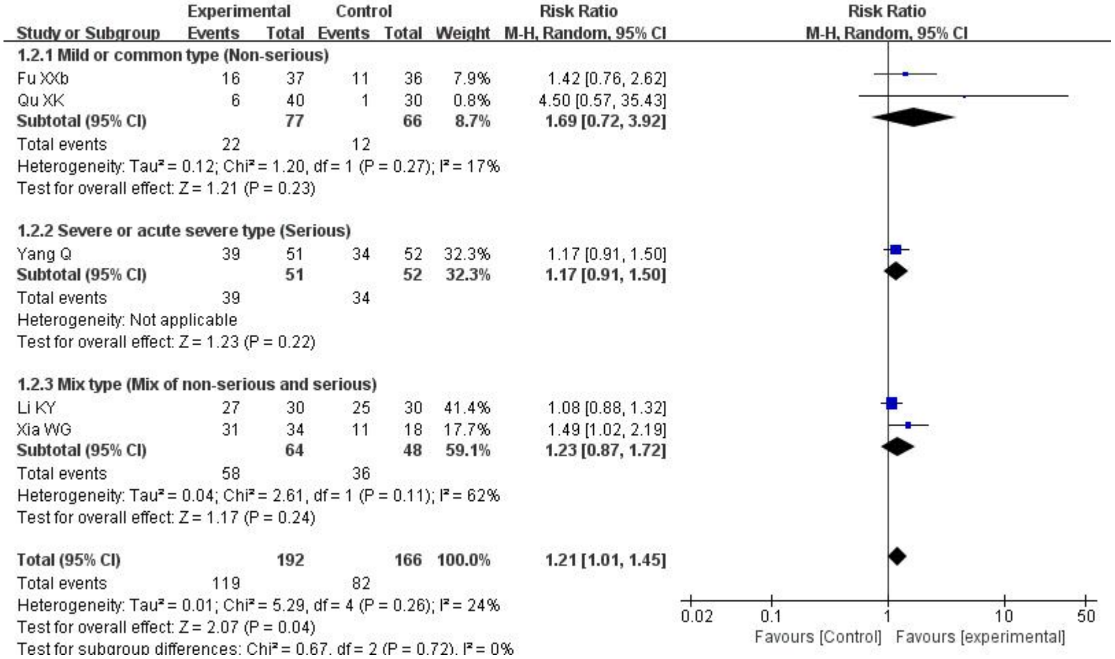

Supplement-Figure 1 Subgroup analysis of Cure rate based on the severity of COVID-19

Study or Subgroup	Experimental Events	Experimental Total	Control Events	Control Total	Weight	Risk Ratio M-H, Random, 95% CI
**1.3.1 Mild or common type (Non-serious)**						
Chen L	3	34	9	34	11.3%	0.33 [0.10, 1.13]
Cheng DZa	4	51	11	51	14.4%	0.36 [0.12, 1.07]
Duan C	9	82	10	41	24.9%	0.45 [0.20, 1.02]
Fu XXa	1	32	3	33	3.4%	0.34 [0.04, 3.13]
Qiu M	0	25	1	25	1.7%	0.33 [0.01, 7.81]
Sun HM	0	32	2	25	1.9%	0.16 [0.01, 3.14]
Wang JB	0	24	2	23	1.9%	0.19 [0.01, 3.80]
**Subtotal (95% CI)**		**280**		**232**	**59.4%**	**0.37 [0.22, 0.63]**
Total events	17		38			
Heterogeneity: Tau² = 0.00; Chi² = 0.77, df = 6 (P = 0.99); I² = 0%						
Test for overall effect: Z = 3.66 (P = 0.0002)						
**1.3.2 Severe or acute severe type (Serious)**						
Ye YA	2	28	1	14	3.1%	1.00 [0.10, 10.11]
**Subtotal (95% CI)**		**28**		**14**	**3.1%**	**1.00 [0.10, 10.11]**
Total events	2		1			
Heterogeneity: Not applicable						
Test for overall effect: Z = 0.00 (P = 1.00)						
**1.3.3 Mix type (Mix of non-serious and serious)**						
Fu XXb	2	37	4	36	6.3%	0.49 [0.09, 2.49]
Li KY	6	30	12	30	23.7%	0.50 [0.22, 1.16]
Xia WG	2	34	6	18	7.5%	0.18 [0.04, 0.79]
**Subtotal (95% CI)**		**101**		**84**	**37.4%**	**0.40 [0.21, 0.79]**
Total events	10		22			
Heterogeneity: Tau² = 0.00; Chi² = 1.49, df = 2 (P = 0.48); I² = 0%						
Test for overall effect: Z = 2.66 (P = 0.008)						
**Total (95% CI)**		**409**		**330**	**100.0%**	**0.40 [0.26, 0.59]**
Total events	29		61			
Heterogeneity: Tau² = 0.00; Chi² = 2.93, df = 10 (P = 0.98); I² = 0%						
Test for overall effect: Z = 4.45 (P < 0.00001)						
Test for subgroup differences: Chi² = 0.68, df = 2 (P = 0.71), I² = 0%						

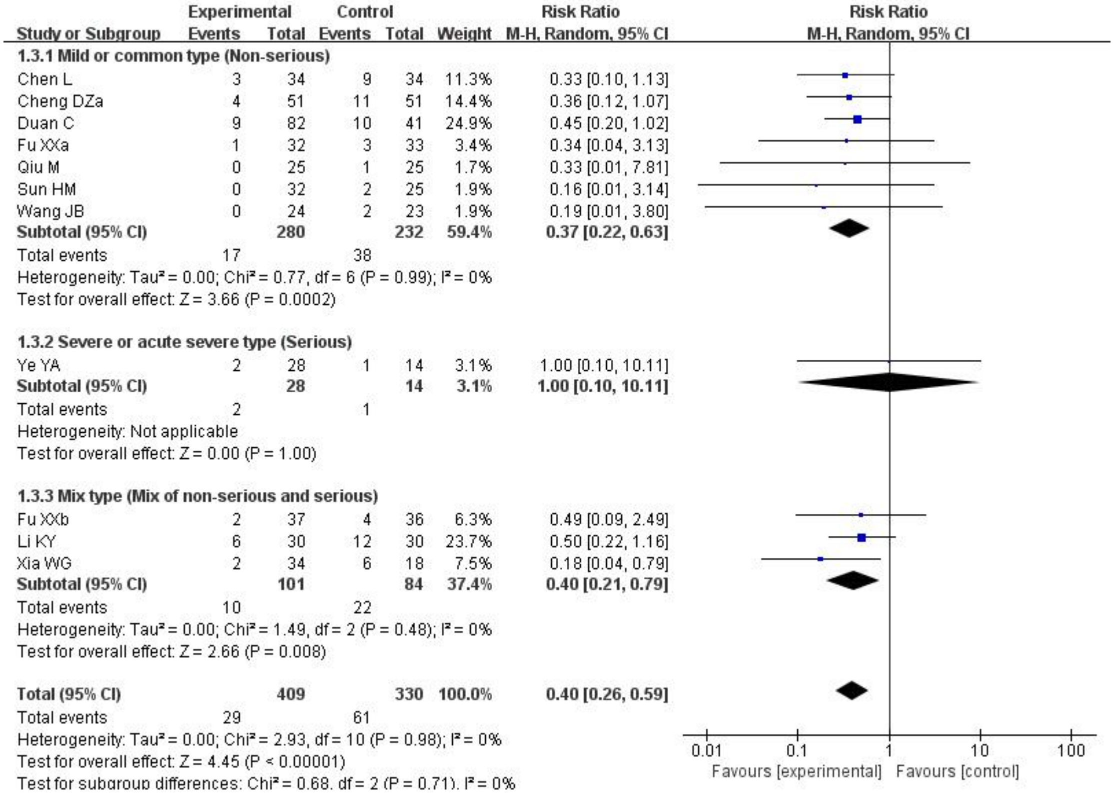

Supplement-Figure 2 Subgroup analysis of the aggravation rate based on the severity of COVID-19

Study or Subgroup	Experimental Events	Experimental Total	Control Events	Control Total	Weight	Risk Ratio M-H, Random, 95% CI
**1.4.1 Mild or common type (Non-serious)**						
Wang JB	0	24	1	23	3.6%	0.32 [0.01, 7.48]
Yu P	1	147	2	148	6.3%	0.50 [0.05, 5.49]
**Subtotal (95% CI)**		**171**		**171**	**9.9%**	**0.43 [0.06, 2.86]**
Total events	1		3			
Heterogeneity: Tau² = 0.00; Chi² = 0.05, df = 1 (P = 0.82); I² = 0%						
Test for overall effect: Z = 0.88 (P = 0.38)						
**1.4.2 Severe or acute severe type (Serious)**						
Yang Q	11	51	16	52	81.6%	0.70 [0.36, 1.36]
Ye YA	1	28	1	14	4.9%	0.50 [0.03, 7.41]
**Subtotal (95% CI)**		**79**		**66**	**86.5%**	**0.69 [0.36, 1.31]**
Total events	12		17			
Heterogeneity: Tau² = 0.00; Chi² = 0.06, df = 1 (P = 0.81); I² = 0%						
Test for overall effect: Z = 1.14 (P = 0.25)						
**1.4.3 Mix type (Mix of non-serious and serious)**						
Xia WG	0	34	1	18	3.6%	0.18 [0.01, 4.23]
**Subtotal (95% CI)**		**34**		**18**	**3.6%**	**0.18 [0.01, 4.23]**
Total events	0		1			
Heterogeneity: Not applicable						
Test for overall effect: Z = 1.06 (P = 0.29)						
**Total (95% CI)**		**284**		**255**	**100.0%**	**0.62 [0.34, 1.14]**
Total events	13		21			
Heterogeneity: Tau² = 0.00; Chi² = 0.96, df = 4 (P = 0.92); I² = 0%						
Test for overall effect: Z = 1.54 (P = 0.12)						
Test for subgroup differences: Chi² = 0.83, df = 2 (P = 0.66), I² = 0%						

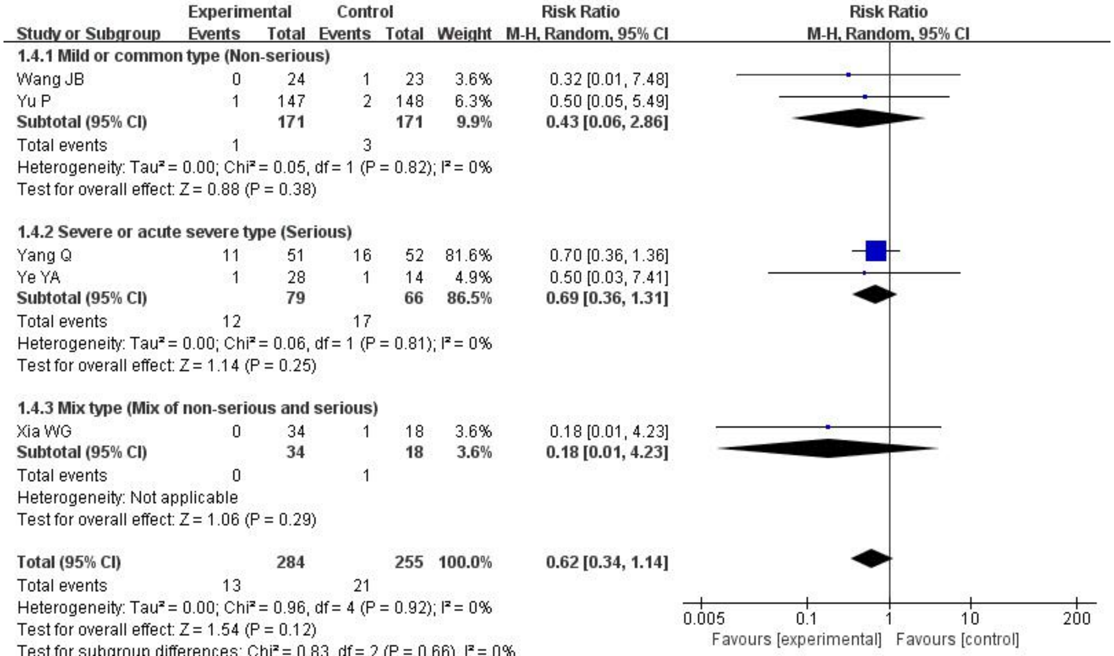

Supplement-Figure 3 Subgroup analysis of the mortality rate based on the severity of COVID-19
